# Engineered multi-functional, pro-angiogenic collagen-based scaffolds loaded with endothelial cells promote large deep burn wound healing

**DOI:** 10.3389/fphar.2023.1125209

**Published:** 2023-03-01

**Authors:** Hengyue Song, Kewa Gao, Dake Hao, Andrew Li, Ruiwu Liu, Bryan Anggito, Boyan Yin, Qianyu Jin, Vanessa Dartora, Kit S. Lam, Lucas R. Smith, Alyssa Panitch, Jianda Zhou, Diana L. Farmer, Aijun Wang

**Affiliations:** ^1^ Center for Surgical Bioengineering, Department of Surgery, UC Davis Medical Center, Sacramento, CA, United States; ^2^ Department of Burns and Plastic Surgery, The Third Xiangya Hospital of Central South University, Changsha, Hunan, China; ^3^ Institute for Pediatric Regenerative Medicine, Shriners Hospitals for Children, Sacramento, CA, United States; ^4^ Division of Plastic Surgery, Department of Surgery, UC Davis Medical Center, Sacramento, CA, United States; ^5^ Department of Biochemistry and Molecular Medicine, UC Davis Medical Center, Sacramento, CA, United States; ^6^ Department of Biomedical Engineering, University of California Davis, Davis, CA, United States; ^7^ College of Biological Sciences, University of California Davis, Davis, CA, United States; ^8^ Department of Neurobiology, Physiology and Behavior, University of California Davis, Davis, CA, United States; ^9^ Department of Physical Medicine and Rehabilitation, UC Davis Medical Center, Sacramento, CA, United States

**Keywords:** deep burn wound, endothelial cell, wound healing, vascularization, ECM scaffold

## Abstract

The lack of vascularization associated with deep burns delays the construction of wound beds, increases the risks of infection, and leads to the formation of hypertrophic scars or disfigurement. To address this challenge, we have fabricated a multi-functional pro-angiogenic molecule by grafting integrin αvβ3 ligand LXW7 and collagen-binding peptide (SILY) to a dermatan sulfate (DS) glycosaminoglycan backbone, named LXW7-DS-SILY (LDS), and further employed this to functionalize collagen-based Integra scaffolds. Using a large deep burn wound model in C57/BLK6 mice (8–10 weeks old, 26–32g, *n* = 39), we demonstrated that LDS-modified collagen-based Integra scaffolds loaded with endothelial cells (ECs) accelerate wound healing rate, re-epithelialization, vascularization, and collagen deposition. Specifically, a 2 cm × 3 cm full-thickness skin burn wound was created 48 h after the burn, and then wounds were treated with four groups of different dressing scaffolds, including Integra + ECs, Integra + LDS, and Integra + LDS + ECs with Integra-only as the control. Digital photos were taken for wound healing measurement on post-treatment days 1, 7, 14, 21, 28, and 35. Post-treatment photos revealed that treatment with the Intgera + LDS + ECs scaffold exhibited a higher wound healing rate in the proliferation phase. Histology results showed significantly increased re-epithelialization, increased collagen deposition, increased thin and mixed collagen fiber content, increased angiogenesis, and shorter wound length within the Integra + LDS + ECs group at Day 35. On Day 14, the Integra + LDS + ECs group showed the same trend. The relative proportions of collagen changed from Day 14 to Day 35 in the Integra + LDS + ECs and Integra + ECs groups demonstrated decreased thick collagen fiber deposition and greater thin and mixed collagen fiber deposition. LDS-modified Integra scaffolds represent a promising novel treatment to accelerate deep burn wound healing, thereby potentially reducing the morbidity associated with open burn wounds. These scaffolds can also potentially reduce the need for autografting and morbidity in patients with already limited areas of harvestable skin.

## 1 Introduction

According to a report published by the World Health Organization (WHO), approximately 180,000 people die worldwide each year from burn injuries, while approximately 11 million people required medical care from burns in 2004 (World Health Organization B, 2018). Since large deep burn wounds (>20% TBSA) often lack the adequate amount of perfused soft tissue to sustain a skin autograft, it requires a greater metabolic effort and more time to develop an appropriate wound bed for autografting (Porter et al., 2016). These contribute to the overall morbidity of large surface area deep burns, which includes insensible fluid and heat losses, increased metabolic demand, increased risk of infection, and overall increased risk of poor scar formation (Gauglitz et al., 2011; Mason et al., 2019). All of this aligns with the WHO’s report, which describes non-fatal burn injuries as severe causes of morbidity, including hospitalization, disfigurement, and disability (World Health Organization B, 2018).

The deep burn wound healing process consists of four phases: haemostasis, inflammation, proliferation, and remodeling. Each of these phases involves different cell types, such as keratinocytes, fibroblasts, endothelial cells, and macrophages (Urciuolo et al., 2019; Vig et al., 2019). Of interest to our study, endothelial cells, keratinocytes, and fibroblasts play the predominant role in the proliferation phase, which includes connective tissue formation, granulation tissue formation, angiogenesis, and epithelialization. Angiogenesis is a prominent feature in proliferation in the wound healing process. This process increases the number of blood vessels and is necessary for the proper delivery of essential oxygen and nutrients through new blood vessels to the wound site, potentially improving the wound healing process. Deep burn wounds with a large surface area, often lack vascularity in the center of the wound, given that the center of burn wounds usually corresponds to the epicenter of the burn zone of injury. Therefore, the center of burn wounds often is dependent on perfusion through the intact blood vessels from the margins of the wound and by diffusion through the uninjured interstitium (Robson et al., 2001; Hunt et al., 2022; Phillips, 2022). Endothelial cells (ECs) lined in the inner layer of blood vessels are an essential cell type for neovascularization. In the center of larger surface area, deep burn wounds, ECs are activated by the hypoxia-driven growth factors such as vascular endothelial growth factor (VEGF) and platelet-derived growth factor (PDGF), breakdown ECM in granulation tissue, proliferate, and migrate and form new capillaries (Eilken and Adams, 2010a). In the remodeling phase, growth factors, matrix metalloproteinases (MMPs), and tissue inhibitors of metalloproteinases (TIMPs) aid in granulation tissue maturation and ECM remodeling. Scar formation is more likely to occur in deep burn wounds and create complications during the deep burn wound healing process. Currently, there are no adequate treatments for deep burn wounds that accelerate wound healing and help prevent the aforementioned complications (Phillips, 2022).

In the clinical setting, burn wound barrier dressings in conjunction with topical antimicrobial agents are utilized as a conservative and traditional method to treat deep burn wounds to physically protect the wound from insensible fluid losses and reduce bacterial bioburden. Allogeneic and xenogeneic skin grafts are also used for wound bed preparation before autografting as another burn wound management strategy to help in cases of large surface area burns that create a severe limitation in the area of donor skin. Modern treatments overcome the deficiency of autografting by utilizing artificial bioengineered dermal templates and scaffolds, such as Integra® and AlloDerm®. Integra, an FDA-approved, biocompatible three-dimensional structure scaffold made from bovine collagen type I crosslinked with chondroitin sulfate from shark cartilage, provides a dressing for vascularization and remodeling in the operative setting for wound treatment. It has been used for over 30 years in burn wound healing, scar and keloid repair, cutaneous tumor reconstruction, giant congenital melanocytic nevus repair, and abdominal reconstruction after necrotizing fasciitis, et. due to its good property of cell migration, proliferation, and blood vessel formation to form neo dermis (Abai et al., 2004; Clayman et al., 2006; Su et al., 2015; Eugénie et al., 2020; Prezzavento et al., 2022). Some have combined this scaffold with cellular elements such as stem cells, keratinocytes, fibroblast, or endothelial cells to accelerate wound healing rate (Kremer et al., 2000; Danner et al., 2012; Banakh et al., 2020; Piejko et al., 2020). However, one of the drawbacks of collagen-based scaffolds is the lack of innate signaling to recruit enough endothelial progenitor cells (EPCs) to promote revascularization (He et al., 2022).

Previous studies identified that LXW7 as a cyclic peptide ligand for the VEGF receptor 2 (VEGF-R2) on ECs and EPCs, which increases VEGF-R2 phosphorylation and activates ERK1/2 mitogen-activated protein kinase (Hao et al., 2017; Olsson et al., 2017; Soldi et al., 2022). Overall, LXW7 shows a strong, stable, and specific ability to retain ECs/EPCs, and provides sufficient endogenous EC recruitment and exogenous EC binding and proliferation. A synthetic collagen-binding peptidoglycan DS-SILY, composed of dermatan sulfate (DS) and collagen-binding peptides SILY was designed to mimic the native decorin, a member of the small leucine-rich proteoglycan (SLRP) family, which consists of a protein core containing leucine repeats with a glycosaminoglycan (GAG) chain consisting of either chondroitin sulfate (CS) or dermatan sulfate (DS) (Stuart et al., 2011). Decorin is related to fibrillogenesis, keloid scar, and hypertrophic scar formation. Recent studies demonstrate that decorin has the ability to prevent collagen from collagenase degradation and decrease scar formation (Khorramizadeh et al., 1999; Armstrong and Jude, 2002; Geng et al., 2006; Mukhopadhyay et al., 2022; Sayani et al., 2022; Scott et al., 2022; Zhang et al., 2022). Therefore, DS-SILY can bind to the collagen scaffold and inhibit collagen degradation.

In this project, we designed a multi-functional collagen-based Integra® scaffold modified by LXW7-DS-SILY (LDS) and loaded with ECs to evaluate the treatment potential benefits in a mouse deep burn wound model. We demonstrate that the LDS modified Integra® not only promotes the ability of proliferation and survival of exogenously seeded ECs, but also accelerates the recruitment of endogenous ECs and angiogenesis in deep burn wounds to build a better-quality wound base and improve wound healing.

## 2 Materials and method

### 2.1 Cell characterization and expansion

C57BL/6 mouse primary bone marrow-derived endothelial cells (ECs) were purchased from Cell Biologics, Inc. (C57-6221). ECs were maintained and cultured in a mouse endothelial cell medium (M1168, Cell Biologics). The passage number of ECs used in all experiments was between passages five and eight in this study. Dil-Ac-LDL staining, immunostaining of CD31 and VE-Cadherin, flow cytometry of CD31, CD34, CD45, CD144, CD90, and tube formation assay were used for mouse EC characterization.

### 2.2 Lentiviral vector transduction

The lentiviral constructs were generated at the University of California, Davis Institute for Regenerative Cures (IRC) Vector Core. ECs were transduced with pCCLc-MNDU3-LUC-PGK-EGFP-WPRE or pCCLc-MNDU3-LUC-PGK-Tomato-WPRE. pCCLc is the backbone of the lentivirus; MNDU3 is a ubiquitous promoter driving the luciferase (LUC) expression. The PGK is another promoter driving the expression of enhanced green fluorescent proteins (EGFP) or td-Tomato fluorescent protein (Tomato). WPRE represents an enhancer that could boost transgene expression. Lentiviral vector was added in transduction media consisting of mouse basal endothelial cell medium, 5% FBS, and 20 μg/ml protamine sulfate (MP Biomedicals) at a multiplicity of infection (MOI) of 10 for 48 h. The transduction medium was then replaced with a complete mouse endothelial cell medium, and the cells were cultured for an additional 72 h.

### 2.3 Acetylated low-density lipoprotein uptake assay

ECs were cultured in serum-free medium for 24 h with the density of 1.2 × 10^4^/well in 24-well plate and then incubated with 10 μg/ml Dil-Ac-LDL (L3484, Invitrogen) for 4 h at 37°C, 5% CO2. Cells were then washed with Dulbecco’s Phosphate buffered saline (DPBS, HyClone) and fixed with 10% formalin (ThermoFisher Scientific) at room temperature (RT) for 5 min, following 3 times wash with DPBS and imaged with a Zeiss Observer Z1 microscope.

### 2.4 Tube formation assay

A 24-well plate was fully covered with 200 μL chilled Matrigel (354234, Corning) per well and incubated at 37°C, 5% CO2 to gel for 1 h 6 × 10^4^ ECs were then seeded onto the Matrigel-coated wells and incubated at 37°C for 18 h. Phase contrast images were taken using a Zeiss Observer Z1 microscope.

### 2.5 Immunofluorescent staining of mouse ECs

5 × 10^4^ ECs were cultured in 24-well plates for 24 h and fixed with 10% formalin for 10 min and blocked with blocking buffer containing 5% bovine serum albumin (BSA, bioWORLD) in 1X DPBS to block non-specific binding sites for 1 h at RT. The cells were then stained with either 0.01 mg/ml PE Rat Anti-Mouse CD31 (553373, BD Biosciences) or 0.01 mg/ml PE Rat Anti-Mouse CD144 (562243, BD Biosciences) antibodies in a staining buffer containing 1% BSA in 1X DPBS and incubated at 4°C overnight. The cell nuclei were stained with 1:5000 DAPI for 5 min and washed 3 times. Then images were taken using a Zeiss Observer Z1 microscope.

### 2.6 Immunostaining analyses of ligand-cell binding affinity

2 × 10^4^/well ECs were cultured in an 8-well cell chamber (80807, ibidi) for 24 h and fixed with acetone at −20°C for 15 min, and non-specific binding sites were blocked with blocking buffer for 1 h at RT. The cells were then stained with 0.001 mg/ml Cy3 Rabbit Anti-Mouse αvβ3 (C02329Cy3, Signalway Antibody) or 1 μM LXW7-FITC or mixed 0.001 mg/ml Cy3 Rabbit Anti-Mouse αvβ3 with 1 μM LXW7-FITC in staining buffer and incubated at 4°C overnight. 1μM LXW7-Scramble-FITC was used as control. The cell nuclei were stained with 1:5000 DAPI for 5 min and imaged using a Nikon A1 confocal microscope.

### 2.7 Attachment and proliferation assay of EC binding on LXW7 modified culture surface

To modify the culture surface with LXW7, a 24-well plate was coated with 150 μL of 10 μg/ml Avidin (Thermo Fisher) and incubated at 37°C for 1 h. Wells coated with Avidin were washed with 1X DPBS 3 times and treated with 150 μL M equivalents (1 μM) LXW7-Biotin and incubated at 37°C for 1 h. D-Biotin was used as a negative control. All treated wells were washed with 1X DPBS 3 times and blocked with blocking buffer at 37°C for 1 h. After all the wells were washed with 1X DPBS 3 times, 1 × 10^5^ ECs were seeded into the wells and incubated at 37°C for 1 h. After incubation, unattached cells were washed with 1X DPBS 3 times. Images were taken by using a Zeiss Observer Z1 microscope. Cell counter ImageJ plugin was used to determine the cell numbers in three randomly selected fields from each independent experiment.

Same as the treatment of the 24-well plate for ligand attachment assay, 96-well plates were coated with 50 μL of 10 μg/ml Avidin and incubated at 37°C for 1 h. After being washed with 1X DPBS 3 times, wells were treated with 50 μL 1 μM LXW7-Biotin or D-Biotin and incubated at 37°C for 1 h then all treated wells were washed and blocked with blocking buffer at 37°C for 1 h 2 × 10^3^ ECs were seeded into each well and cultured at 37°C for the next 7 days. MTS assay (G3580, Promega) was performed following the manufacturer’s instructions. Absorbance was measured at 490 nm and 630 nm using a SpectraMax i3 plate reader instrument (Molecular Devices LLC).

### 2.8 Synthesis and characterization of peptide-hydrazides

Hydrazide-modified peptides RRANAALKAGELYKSILYGSG-hydrazide (SILY-hydrazide) and cGRGDdvc (AEEA)2WG-hydrazide (LXW7-hydrazide, wherein AEEA is short PEG linker) were synthesized using standard Fmoc solid-phase peptide synthesis following previously established protocols with modification (Paderi and Panitch, 2008; Xiao et al., 2010). In brief, Cl-TCP(Cl) ProTide Resin (loading 0.4–0.6 mmol/g, CEM Corporation) was rinsed three times with dichloromethane (DCM, Fisher Scientific) and N, N-Dimethylformamide (DMF, Fisher Scientific) and expended in 50% DCM/DMF for 1 h. As soon as the resin was swollen, it was reacted twice with 10% hydrazine hydrate (Sigma) in DMF and 0.057MN, N-Diisopropylethylamine (DIPEA, Fisher Scientific) for 2 h at RT each. To cap any unreacted chloride groups, 10% methanol (Fisher Scientific) in DMF was used, and the resin was then washed three times in DMF, as well as 3 times in DCM. After this, the resin was treated with four equivalents of the first Fmoc-amino acid, four equivalents of OxymaPure, N,N-diisopropylcarbodiimide (DIC, Fisher Scientific), and ten equivalents of DIPEA in DMF for 4 hours, followed by three repetitions of the wash. Using a Liberty Blue microwave peptide synthesizer (CEM Corporation), subsequent amino acids were coupled for 10 min each at 50°C with 5 equivalents of Fmoc-amino acids, DIC, and OxymaPure containing 0.1 M DIPEA. The deprotection process was carried out using 20% piperidine in DMF. To cleave peptides off beads, this reaction was conducted using trifluoroacetic acid (TFA, Fisher Scientific), phenol (Sigma), and water (5% H2O) for 3 h. Fresh peptides were precipitated using cold diethylether (Acro Organics) and then allowed to dry before dissolving in 5% acetonitrile/water for purification. The cysteine residues in LXW7 were first oxidized using ClearOx resin (Peptides International) in accordance with the manufacturer’s guidelines before purification. Using an AKTApure 25 FPLC (GE Healthcare) and an acetonitrile gradient, peptides were purified through a C18 prep column against an acetonitrile gradient and verified using MALDI-TOF-MS (Bruker). Peptides were purchased from InnoPep Inc. for some experiments.

### 2.9 Synthesis and characterization of molecule variants

As described in our previous studies (Walimbe et al., 2021), LDS was synthesized by conjugating SILY-hydrazide and LXW7-hydrazide to a dermatan sulfate (DS) backbone using carbodiimide chemistry. DS (average molecular weight 41,816 Da, Celsus Laboratories) was reacted with peptide-hydrazides by 1-ethyl-3-[3-dimethylaminopropyl] carbodiimide hydrochloride (EDC, ThermoFisher Scientific) in 0.1 M MES [2- (N-morpholino) ethanesulfonic acid] buffer with 8 M urea (Sigma) and 0.6% NaCl (Sigma) titrated to pH 4.5. First, SILY-hydrazide was conjugated to the DS for 4 h using 0.01 mM EDC. To stop the reaction, pH eight was titrated. A tangential flow filtration (Spectrum labs) was used to purify the product with a 10 kDa column prior to lyophilization. In a similar manner, 0.1 mM EDC was used to sequentially conjugate LXW7-hydrazide to DS-SILY constructs for 24 h prior to purification. To verify peptide conjugation, standard curves were created using concentration-dependent 280 nm absorbance of aromatic amino acids, and absorbances of synthesized molecules were extrapolated using readings obtained on a NanoDrop UV–Vis spectrophotometer (Thermo Fisher).

### 2.10 Preparation of LDS-modified integra scaffold with or without ECs

6 mm diameter punch-outs of Integra were cut using a sterile biopsy punch and placed into two 35 mm dishes with the collagen side facing up and incubated with 20 μL of 10 μM LDS or 1X DPBS at 37°C for 1h. The scaffold was washed 3 times with 1x DPBS and soaked in the ECs culture medium at 37°C for 1h. The scaffold was then placed separately into each well of the 48-well plate. 1 × 10^6^ cells/cm2 ECs were suspended in 20 μL of ECs culture media per scaffold and carefully loaded onto the surface of LDS-modified Integra and unmodified Integra. The plate was placed in a 37°C, 5% CO2 incubator, incubated for 1h for cells to adhere to the scaffold, and then added 100 μL culture media into each well.

To prepare the scaffold for surgery, 2 × 3 cm^2^ Integra was cut by sterile scissors, placed in 35 mm dishes separately with the collagen side facing up, and incubated with 500 μL of 10 μM LDS or 1X DPBS for 1h at 37°C. The scaffold was rinsed three times with DPBS and soaked in the culture medium at 37°C for 1h 1 × 10^6^ cells/cm^2^ ECs were suspended in 200 μL of ECs culture media per scaffold and carefully loaded onto the surface of LDS-modified Integra and unmodified Integra. Then scaffolds were cultured for 24h at 37°C, 5% CO2 incubator prior to surgery.

### 2.11 Attachment and proliferation assay of EC binding on LXW7 modified collagen base scaffold

To further explore the effect of LXW7 on mouse EC binding efficacy on a collagen-based scaffold, 1 × 10^6^ cells/cm^2^ mouse ECs were seeded on the LDS-modified or untreated 6 mm diameter Integra scaffold and incubated for 1 h. After incubation, unattached cells were washed off with 1X DPBS 3 times. Images were taken using a Nikon A1 confocal microscope. Cell counter ImageJ plugin was used to determine the cell numbers in three randomly selected fields from each independent experiment.

A cell proliferation assay was performed using 1 × 10^6^ mouse ECs/cm^2^ seeded on the LDS modified or untreated 6 mm diameter Integra scaffold and incubated for 5 days. MTS assay was performed following the manufacturer’s instructions. Absorbance was measured at 490 nm and 630 nm using a SpectraMax i3 plate reader instrument (Molecular Devices LLC).

### 2.12 Mouse large deep burn wound healing model

All animal procedures were approved by the University of California, Davis (UCD) Institutional Animal Care and Use Committee (IACUC). Male C57BLK/6 mice (Jackson Lab, 8–10 weeks old) were used in this project. The dorsal hair of the mice was shaved and cleaned with 70% ethanol right before burn injury creation. Mice were anesthetized with 3% isoflurane and placed dorsal skin exposed in a 2 cm × 3 cm window of a rack. Full-thickness skin burn wound was created by immersing the bottom of the rack in 65°C water for 20 s (Nguyen et al., 2022; Shen et al., 2022). 48 h after the burn wound injury, the burned skin (2 cm × 3 cm) was removed and different groups of scaffolds, including Integra only, Integra + ECs, Integra + LDS and Integra + LDS + ECs were placed on the wound area. Buprenorphine (0.03 mg/mouse) and 0.9% saline (1 mL) was given intraperitoneally for analgesia and fluid resuscitation immediately after injury. On days 1, 7, 14, 21,28, and 35 post-treatments, macroscopic photos were taken of all wounds for further measurements.

### 2.13 Histological analyses

Animals were euthanized at two time points, day 14 and day 35 after treatment. The 2 cm full-thickness wound skin tissue samples were collected within the wound area at the center of each tissue sample. All tissue samples were fixed in 4% paraformaldehyde for 24 h, dehydrated in 30% sucrose for 48 h, embedded in O.C.T. compound (Sakura Finetek USA), and stored in −80°C. 10μm thickness sections were cut and prepared by the Cryostat (Leica CM3050S). Hematoxylin and Eosin (H&E) staining was performed to observe the wound tissue formation. Masson Trichrome (22110648, Epredia™) staining was used to evaluate the collagen deposition. All the images were captured and analyzed by the 4x lens of ImageXpress Pico Automated Cell Imaging System (Molecular Devices). Picro Sirius Red staining (ab245876; Abcam) was performed to observe the different collagen alignment in the wound area at different timepoints. Polarized images were captured by 10x lens of Leica DMi8 microscope under linearly polarized light by inserting a rotating polarizer into the beam path before and after the section, respectively. Once Picro Sirius Red staining images were captured, they were processed in MATLAB for analysis and graphing (Rich and Whittaker, 2017).

For immunostaining images, tissue sections were washed with 1X DPBS, permeabilized with 0.5% Triton X-100 for 10 min, blocked with blocking buffer for 1h, and stained by the following primary antibodies by incubating in 4°C overnight: PECAM-1(1:200, goat, AF3628, R&D Systems), α-SMA (1:200, rabbit, ab5694, abcam). Sections were then incubated with their respective secondary antibodies diluted at 1:500 for 1 h, counterstained with DAPI (1:5000) for 5 min, and mounted with Prolong Diamond Antifade Mountant (P36961, Invitrogen). Nikon A1 laser-scanning confocal microscope was used to acquire images. The number of blood vessels (α-SMA positive) per field was counted.

### 2.14 Statistics

Data are reported as mean ± standard deviation (SD) for cell attachment and MTS assay and as mean ± standard error of mean (SEM) for healing rate and histological analysis. Statistical analysis of cell attachment assay was performed using unpaired two-tailed distribution, equal variance Student’s t-test. Analyses of histological analysis including wound length, re-epithelialization, collagen volume fraction and number of blood vessels were performed using one-way ANOVA. MTS assay and Picro Sirius Red Staining was performed by two-way ANOVA. Healing rate was performed by mixed-effects analysis. All statistical analyses were performed using PRISM 8 (GraphPad Software Inc.), and differences were considered significant when *p* < 0.05.

## 3 Result

### 3.1 Characterization and transduction of mouse bone marrow ECs

**FIGURE 1:**
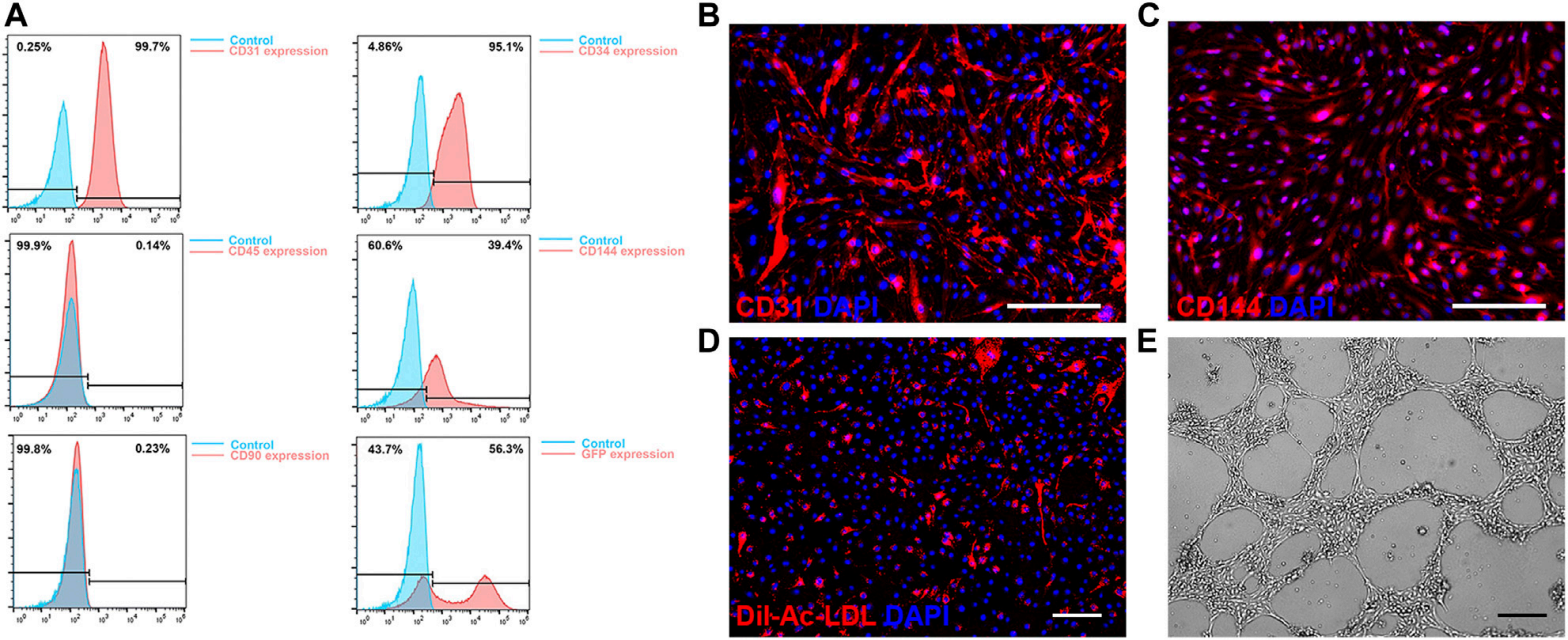
Characterization of mouse bone marrow derived endothelial cells (ECs). **(A)** Flow cytometry of CD31, CD34, CD45, CD144 and CD90 expression and GFP transduction on ECs. **(B, C)** Immunofluorescence staining results of expression of CD31 **(B)** and VE-Cadherin **(C)**. **(D)** Acetylated low-density lipoprotein uptake by ECs. **(E)** Representative phase contrast image of *in vitro* tube formation of ECs. Scale bar = 200 μm.

ECs derived from mouse bone marrow exhibited typical EC morphology and were efficiently transduced with the lentiviral vector expressing fluorescent marker GFP for analysis. A significant positive expression of CD31, CD34, CD144, as well as a negative expression of CD45 and CD90 in flow cytometry, demonstrated the EC characteristics (Figure 1A). A high level of expression of EC markers CD31 and CD144 in immunofluorescence staining verified their EC characteristics (Figures 1B, C). In mouse ECs co-cultured with DiI-Ac-LDL, positive staining for DiI-Ac-LDL was observed (Figure 1D). *In vitro* tube formation assays demonstrated their ability to form tubules in the presence of basement membranes (Figure 1E). Based on these results, mouse bone marrow derived ECs and human ECs have similar phenotypes and functions.

### 3.2 LXW7 ligands accelerated mouse EC attachment and proliferation

**FIGURE 2:**
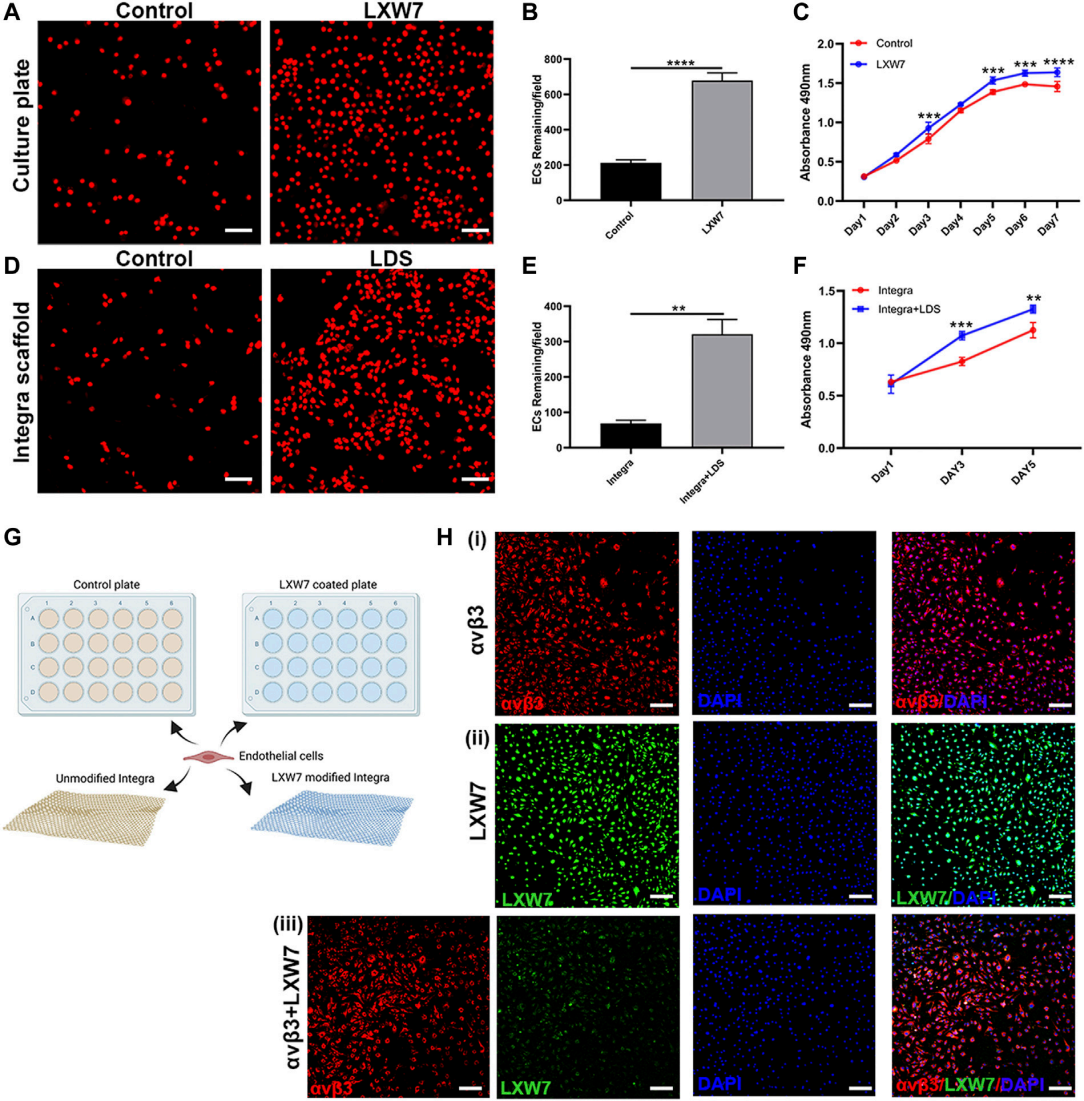
Effects of LXW7 and LDS on the attachment and proliferation of ECs. **(A, D)** Representative images of ECs attached on D-Biotin(control) or LXW7-Biotin treated culture plate and Integra (control) **(A)** or LDS treated Integra scaffold **(D)** after 30 min incubation. **(B, E)** Quantitative data and analysis of remaining cells on culture plate **(B)** and Integra scaffold **(E)**. **(C, F)** Proliferation and viability of ECs on D-Biotin (control) or LXW7-Biotin treated culture plate **(C)** and on Integra scaffold or LDS treated Integra scaffold **(F)** were measured by MTS assay at different timepoints. **(G)** Scheme of design of ECs attachment assay on culture plate or Integra scaffold. **(H)** Representative images of integrin αvβ3 expression on ECs (i), LXW7 binding efficiency on ECs (ii), and integrin αvβ3+LXW7 co-staining (iii). Scale bar = 50 μm. Data are expressed as mean ± SD. *p < 0.05, **p < 0.01, ***p < 0.001, and ****p < 0.0001.

To demonstrate the abilities of LXW7 to enhance mouse EC attachment and proliferation, we tested the cell-ligand binding ability in different situations with tissue culture plate and collagen-based scaffold Integra. To modify the tissue culture plate surface, we used LXW7-Biotin as treatment and D-Biotin as a negative control. To further explore the ability of cell-ligand binding ability on scaffold, we modified the Integra with LDS to allow the LXW7 to functionalize in the scaffold. After seeding mouse ECs on the tissue culture plate and collagen-based Integra scaffold, our results demonstrated that LXW7 can aid in the attachment of mouse ECs to the tissue culture surface, while LDS can accelerate the attachment of mouse ECs to the scaffold (Figures 2A, D, G). The remaining cells on the modified plate and Integra were higher than those on the plate and Integra without LWX7 or LDS treatment (Figures 2B, E).

To further clarify the ability of proliferation, we performed the MTS assay both on tissue culture plate surface and collagen-based Integra scaffold. After incubating mouse ECs on LWX7-Biotin or D-Biotin treated tissue culture plate for 7 days, we observed that the cell proliferation rate was rapid from Day 1 to Day 5, while from Day 5 to Day 7 the rate decreased. Compared to the D-Biotin treated group, the LXW7 treated surface significantly enhanced cell proliferation from Day 2 (Figure 2C). After incubating mouse ECs on collagen-based scaffold for 5 days, we observed the same trend as the tissue culture plate, that LDS modified Integra can improve mouse EC proliferation (Figure 2F). The results clearly demonstrate that the LXW7 modified surface and LDS-functionalized scaffold are effective in promoting mouse EC attachment and proliferation.

To clarify the ligand-cell binding affinity, we stained mouse ECs with integrin αvβ3 antibody and LXW7 to evaluate the ligand binding to the mouse ECs through integrin αvβ3 (Figure 2H). Mouse ECs showed high expression of integrin αvβ3 (Figure 2Hi). LXW7 also showed high binding efficiency on ECs. After incubating integrin αvβ3 antibody and LXW7 with mouse ECs at the same time, LXW7 colocalized with the integrin αvβ3 on the mouse ECs, suggesting that LXW7 has high binding efficiency to mouse ECs through integrin αvβ3.

### 3.3 LDS accelerated the wound healing rate in the mouse large deep burn wound model

**FIGURE 3:**
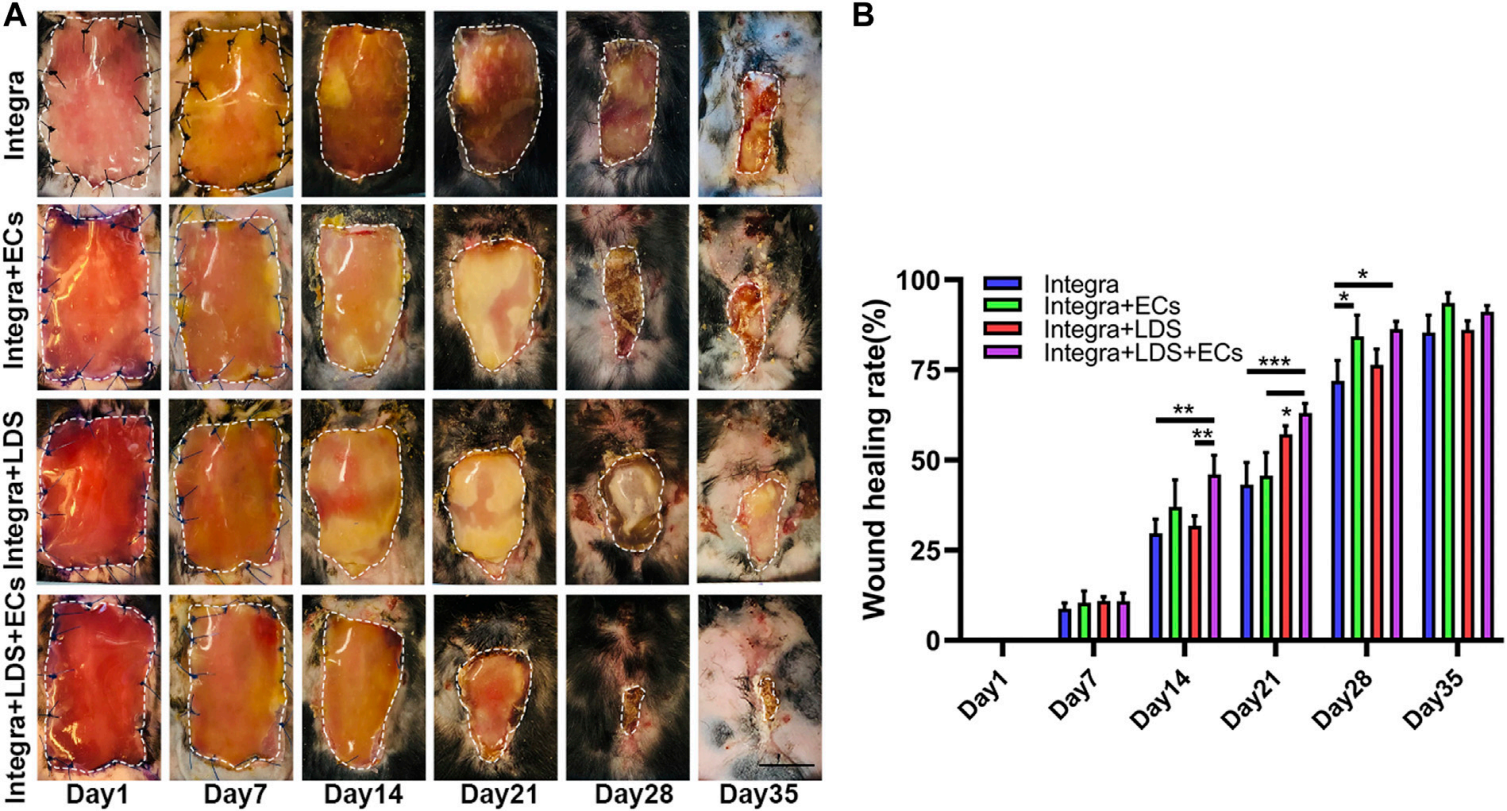
Characterization of wound healing in large deep burn wound model. The wounds were treated with Integra with ECs, Integra with LDS, or Integra with ECs and LDS, or the Integra only as standard of care. **(A)** Representative images of wounds in all groups during healing over 35 days. Relative wound area is indicated with white dotted line. Scale bar = 1 cm. **(B)** Quantitative data of wound healing rate of four groups at different time points. Data are expressed as mean ± SEM. *p < 0.05, **p < 0.01, and ***p < 0.001.

The large deep burn wound model was used to evaluate the effect of different treatment groups on large deep burn wounds *in vivo*. The group compositions are as follows: Integra only (negative control); Integra + ECs; Integra + LDS; Integra + LDS + ECs. Digital photos were taken at Days 1, 7, 14, 21, 28, and 35 (Figure 3A). The wound area decreased in all groups over time. The average wound healing rate was significantly increased at week two in Integra + LDS + ECs (46.049% ± 5.3%) compared to both Integra (29.678% ± 3.925%) and Integra + LDS (31.78% ± 2.744%). In week 3, the wound healing rate was accelerated in Integra + LDS + ECs (63.018% ± 2.65%) compared to both Integra (43.186% ± 6.153%) and Integra + ECs (45.687% ± 6.443%). In week 4, the wound healing rate was higher in Integra + LDS + ECs (86.253% ± 2.176%) and Integra + ECs (84.33% ± 5.853%) in comparison to Integra (71.928% ± 5.671%). On Day 35, there was no significant difference among four groups (Figure 3B). Overall, Integra + LDS + ECs shows great potentials in the proliferation and early remodeling stage of large deep burn wound healing.

### 3.4 LDS accelerated deep burn wound re-epithelialization and decreased wound length

**FIGURE 4:**
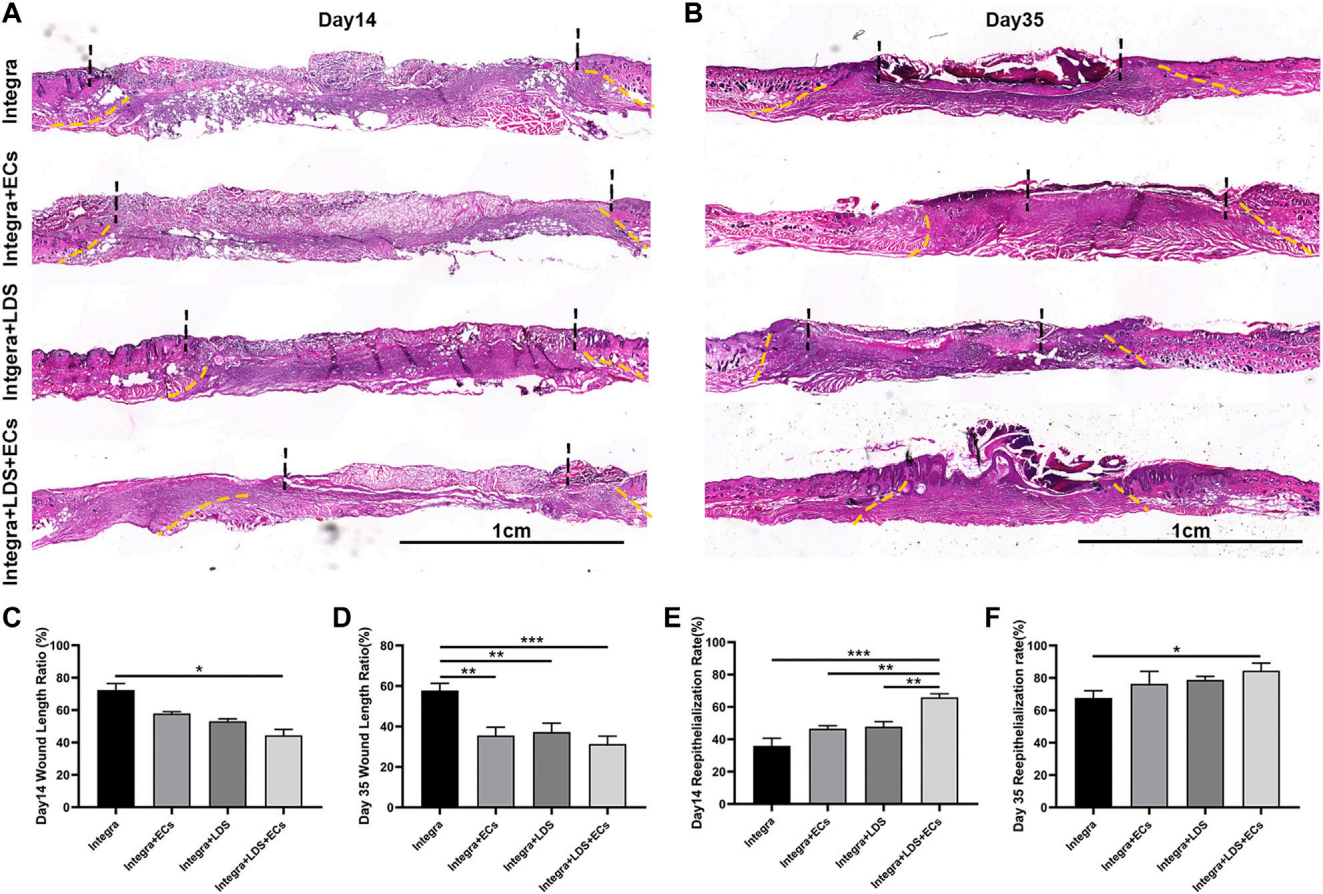
Histological evaluation of wound regeneration at Day 14 and Day 35. **(A,B)** Hematoxylin and eosin (H&E) staining was performed at Day 14 and Day 35 post-wounding for four groups. The wound area and normal tissue were separated by yellow dot lines. The un-epithelialized area and re-epithelialized area were separated by black dot lines. Scale bar = 1 cm. **(C,D)** Quantification of the wound length at Day 14 and Day 35. **(E,F)** Quantification of the re-epithelialization at Day 14 and Day 35. Data are expressed as mean ± SEM. *p < 0.05, **p < 0.01, and ***p < 0.001.

To further evaluate the wound healing qualities at different time points, H&E staining was performed on large deep burn wound tissue at Day 14 and Day 35 (Figures 4A, B). It showed that Integra + LDS + ECs had the shortest wound length among all groups, followed by Integra + ECs, Integra + LDS and Integra groups at Day 35 (Figure 4E). At Day 14, the same trend occurred among these groups. The Integra + LDS + ECs group exhibited the shortest wound length in all groups. And Intgra + ECs showed longer wound length compared to Integra + LDS + ECs. Integra only showed longer wound length in Day 14 and Day 35 among four groups (Figure 4C). Also, the Integra + LDS + ECs was fully covered with neo epidermis and showed significantly better re-epithelialization out of all four groups, while the Integra + ECs, Integra + LDS, and Integra only groups were partially covered with neo epidermis at Day 35 (Figure 4F). At Day 14, the Integra + LDS + ECs group showed the trend of improved re-epithelialization among all groups (Figure 4D). Integra only showed more un-epithelialized area in Day 14 and Day 35. Overall, the Integra + LDS + ECs group has better wound healing qualities at Day 14 and Day 35.

### 3.5 LDS modified integra scaffold increased collagen deposition and resulted in optimal collagen composition

**FIGURE 5:**
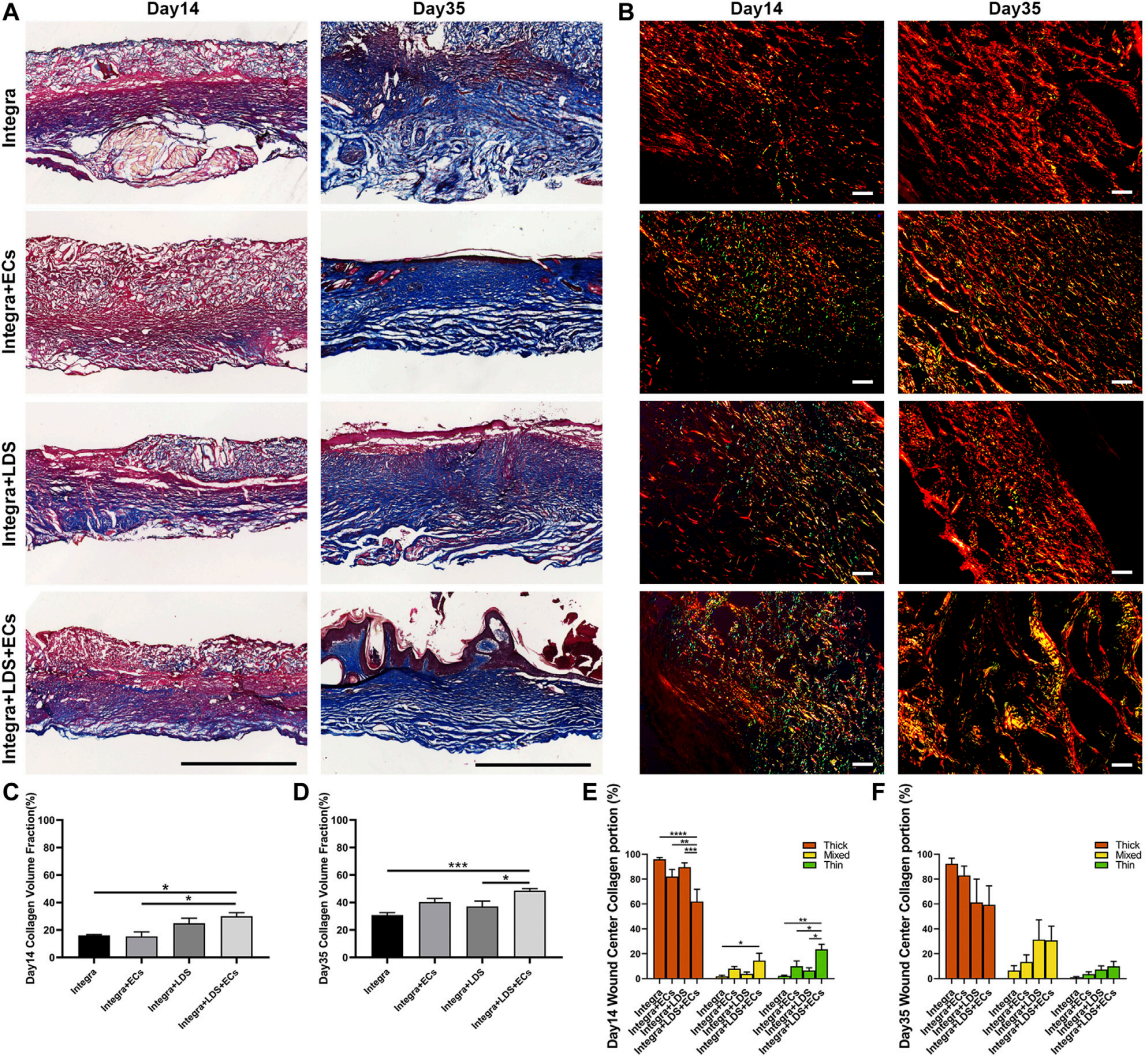
Histology evaluation of collagen deposition and portion of wounds treated by different groups at Day 14 and Day 35. **(A)** Representative images of Masson Trichrome staining at Day 14 and Day 35. Collagen stains blue, nuclei stain dark brown, whereas muscles, cytoplasm, and keratinocyte stain red. Scale bar = 1 cm. **(B)** Representative images of Picro Sirius Red staining under linear polarized light microscope at Day 14 and Day 35. Red birefringence represents thick fibers (Type I), while green birefringence represents thin fibers (Type III). Yellow birefringence represents mixed fiber. Scale bar = 20 μm. **(C, D)** Quantification of the collagen volume fraction at Day 14 and Day 35. **(E, F)** Quantification of the percentage of collagen type of total collagen at Day 14 and Day 35. Data are expressed as mean ± SEM. *p < 0.05, **p < 0.01, and ***p < 0.001, and ****p < 0.0001.

Masson trichrome and Picro Sirius Red staining were conducted on large deep burn wound samples at Day 14 and Day 35 in order to evaluate the quality of collagen in different groups at different time points (Figures 5A, B). Collagen stains blue, nuclei stain dark brown, whereas muscles, cytoplasm, and keratinocyte stain red. In all groups, Masson Trichrome staining showed that newly formed collagen was arranged in the regenerated tissue at Day 14 and Day 35. A typical collagen fiber, with densely packed and basket-weave patterns, was observed at Day 14 for Integra + LDS and Integra + LDS + ECs groups; and at Day 35 for Integra + LDS + ECs and Integra + ECs groups. At Day 14, the collagen deposition of Intgera + LDS + ECs was accelerated compared to the Integra only and Integra + ECs groups (Figure 5C). At Day 35, the collagen volume fraction of Integra + LDS + ECs was significantly higher than the Integra only and Integra + LDS groups (Figure 5D). Picro Sirius Red staining showed different collagen types in deep burn wounds at different time points. The red birefringence represents thick fibers (Type I), while the green birefringence represents thin fibers (Type III). The yellow birefringence represents mixed fiber, which is the combination of collagen I and collagen III. The type and amount of collagen change during wound healing, which determines the tensile strength of skins. In the early stages of wound healing, collagen III is synthesized first, followed by collagen I, the dominant type of collagen in the skin. As shown in Figure 5E, at Day 14, the proliferation stage of wound healing, the Integra + LDS + ECs group induced significantly higher collagen III deposition compared to the other three groups. Also, the mixed collagen in the Integra + LDS + ECs treated group was higher than Integra only group. At Day 35, the main portion of collagen in the wound was collagen I. The Integra + LDS + ECs group and the Integra + LDS group showed the trend of increasing mixed collagen compared to other two groups (Figure 5F). These results indicated that the Integra + LDS + ECs group showed promising results in collagen deposition, collagen proportion and potentially decreased scar formation.

### 3.6 LDS promoted angiogenesis in the large deep burn wound model

**FIGURE 6:**
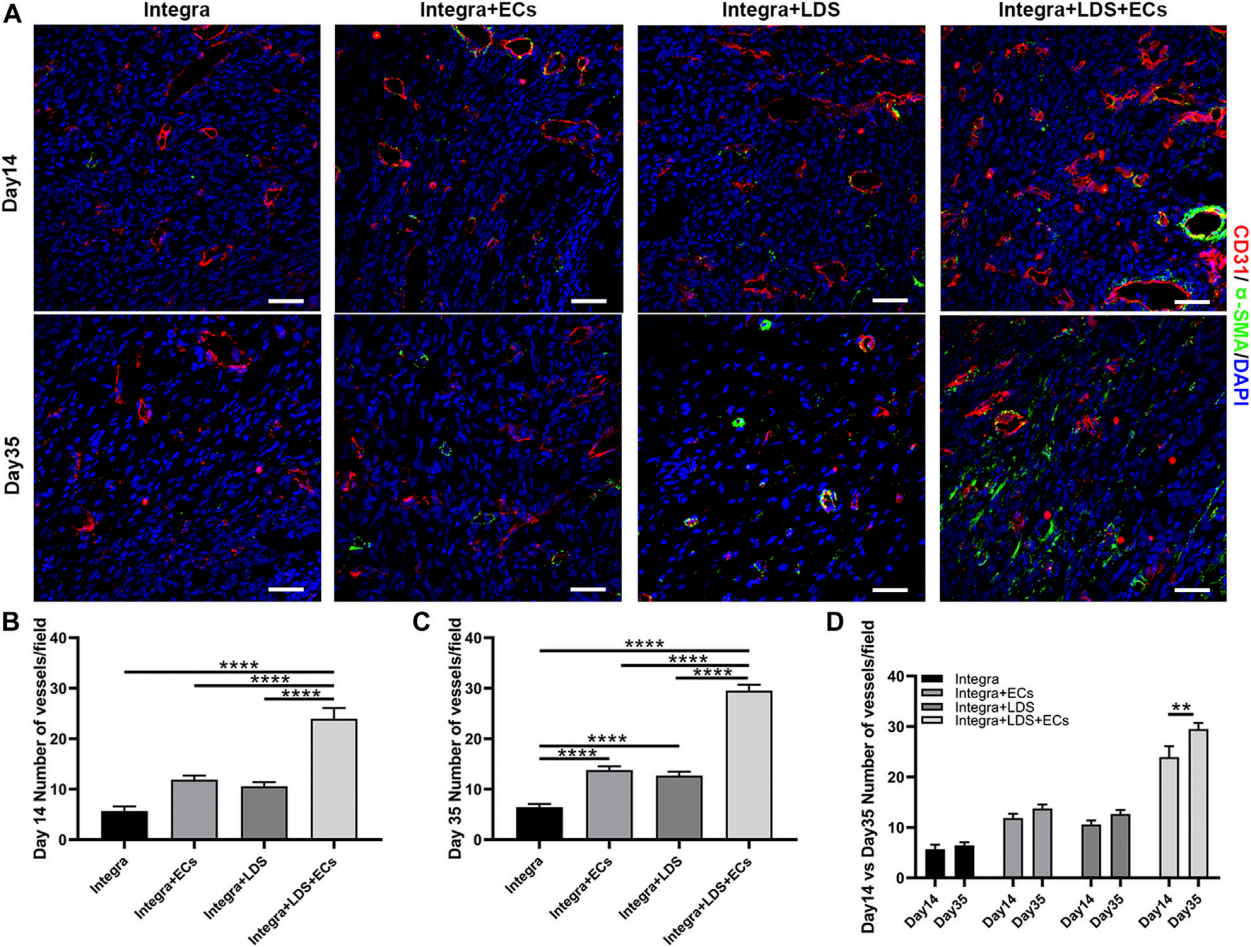
Immunofluorescence staining of CD31 and α-SMA in wound samples on Day 14 and Day 35. **(A)** Representative images of CD31 and α-SMA co-immunostaining in all groups at Day 14 and Day 35. Scale bar = 50 μm. **(B–D)** Quantitative data of newly formed vessels per field on Day 14 and Day35. Data are expressed as mean ± SEM. *p < 0.05, **p < 0.01, ***p < 0.001, and ****p < 0.0001.

To measure the angiogenesis in large deep burn wound at different time points, we stained deep burn wound tissue with CD31 and α-SMA to evaluate the number of blood vessels in the wound center at Day 14 and Day 35 (Figure 6A). At Day14 and Day 35, the Integra + LDS + ECs group showed higher blood vessel number than other three groups. Integra + LDS and Integra + LDS + ECs group showed a significantly higher number of blood vessels compared to Integra group at Day 35 (Figures 6B, C). Only Integra + LDS + ECs group indicated the increase of blood vessels from Day 14 to Day 35 (Figure 6D). In addition, the blood vessels in Integra + LDS + ECs group show more mature and completed structures. Integra + LDS + ECs showed the highest CD31 density among all groups, followed by Integra + ECs, Integra + LDS and Integra groups at Day 14. Also, CD31 in all four groups exhibited a from Day 14 to Day 35.

## 4 Discussion

The wound healing process involves various cell types, cytokines, and chemokines and consists of four overlapping phases: homeostasis, inflammation, proliferation, and remodeling. To further explore how LDS performs on a large wound model, we created large deep burn wound area on the dorsal of C57 BLK6 mice. The wound area is about 20 times larger than a 6 mm punch wound. Unlike normal wound healing, large deep burn wound has a unique and complex healing process. Thermal injury potentially increases the damage to the area after injury, causing hypoxia, damage of blood vessels, and skin tissue loss. Due to the large amounts of tissue loss in the deep burn wound area, these complex wounds lead to insensible fluid and heat loss, higher infection rate, prolonged time for wound bed preparation before autografting, and increasing need for skin autografting. These can lead to suboptimal scar formation that can be painful and eventually lead to debilitating contractures that require long term physical therapy.

One of the key processes in deep burn wound healing is rebuilding the vasculature network in the wound bed to deliver oxygen, nutrients, and other biological moieties to allow cellular proliferation, migration, and tissue regeneration. Endothelial cells serve a critical role in efficient vascularization in wound healing. Activated ECs break down ECM in the granulation tissue, proliferate, migrate, form new cell-cell junctions, and branch out to form new capillaries (van Hinsbergh and Koolwijk, 2008; Vestweber, 2008; Dejana et al., 2009; Arroyo and Iruela-Arispe, 2010).

LXW7, discovered by screening OBOC libraries, has a high binding affinity to αvβ3 integrin on ECs/EPCs. In our previous study, due to increased phosphorylation of VEGF receptor 2 (VEGF-R2) and activation of ERK1/2, LXW7 helps ECs/EPCs proliferation, migration, and recruitment (Hao et al., 2017). Meanwhile, *in vitro* data supports that mouse ECs have highly improved attachment and proliferation abilities on both LXW7 treated culture plate and LDS treated Integra scaffold (Figure 2), which shows that scaffolds treated with LDS have the potential to help EC survive, proliferate and attach. On Day 14, both Integra + ECs and Integra + LDS + EC groups show higher wound healing rates compared to the other two groups, indicating exogenous ECs are essential to the large deep burn wound at the early stage. *In vivo*, the ability of wound healing arose in group Integra + LDS on Day21, which indicates that the LXW7 potentially recruits endogenous ECs to the wound site to accelerate angiogenesis and wound healing. Because of the lack of blood supply, it takes time for adequate numbers of endogenous ECs to migrate to the wound site and take effect in the neovascularization process. LXW7 cooperated with exogenous ECs to aid in proliferation, retention, and functionalization at the wound site. Some studies have shown that EC transplantation may benefit skin wound healing by promoting the recruitment of monocytes/macrophages and increasing neovascularization at the wound site (Suh et al., 2005). The binding of growth factors to proteoglycans and, thereby, physical linkage to matrix scaffolds is essential for the generation of growth factor gradients, which are critical for filopodia extension and the directional growth of endothelial sprouts in neovascularization (Ruhrberg et al., 2002; Gerhardt et al., 2003; Eilken and Adams, 2010b). IHC shows that on Day 35, the number of endothelial cells decreased. Of interest is that the signal of transplanted ECs disappeared on day 35. This may have been because, during the remodeling phase, the cellular processes activated in the acute phase following injury are downregulated and eventually halted. There are few cells in the wound bed, consisting primarily of collagen and other proteins that make up the extracellular matrix, as endothelial cells, macrophages, and myofibroblasts undergo apoptosis or migrate out of the affected area (Gurtner et al., 2008). Another interesting observation is that on the Day35, the Integra + ECs and Integra + LDS + ECs groups showed better-wound healing, though there was no significant difference between the four groups. Some studies posit that angiogenesis is a critical determinant of wound healing (Arnold and West, 2022; Folkman, 2022; Swift et al., 2022), while others, like Jacobi, J. observed that Wound healing was not affected by the same degree of impairment in wound angiogenesis (Jacobi et al., 2004). It is important to note that in the large deep burn wound, wound closure is not a consistently used endpoint for wound healing. After wound closure, the remodeling and maturation often continue for months to years. As such, it is especially important to determine how to optimize the remodeling phase to minimize scarring.

We accomplished this by studying not only vascularization but also collagen deposition. We used Day 14 and Day 35 to represent time points within the proliferation and remodeling phases, respectively (Baldassarro et al., 2022). In a previous study, DS-SILY was shown to decrease collagen degradation by inhibiting MMP, thus, reducing dermal scarring (Stuart et al., 2011). Quantification in Masson Trichrome staining on Day 14 in Figure 5C indicates that at an early stage of collagen formation, DS-SILY may offer better support for collagen from MMP degradation. The collagens form a relaxed network of cross-linked long-chain fibers to give the strength and elasticity of healthy skin and scar tissue. The two dominant types of collagens in wound repair are collagen I and III. Remodeling involves the active remodeling of the acellular matrix from one primarily made up of type III collagen to one primarily composed of type I collagen (Lovvorn et al., 2022). It is known that, the ratio of collagen I to collagen III is essential in scar formation. In hypertrophic scar and keloid, this ratio is often perturbed, arising up to 17:1, almost 3 times higher than in normal skin (6:1) (Verhaegen et al., 2009; Wolfram et al., 2009; Xue and Jackson, 2015; Uitto J Fau - Perejda et al., 2022). Studies have shown that collagen III is essential for the non-scar healing process (Liu et al., 2013; Wang et al., 2018; Wang et al., 2019). At Day 14, the collagen III content in the wound model was higher in the Ingtegra + LDS + ECs group, also at Day 35, the portion of mixed collagen I and III was higher in the Integra + LDS + ECs and the Integra + LDS groups, suggesting large deep burn wounds treated with LDS with ECs may have formed less scarring. Unlike human skin, rodent animal has a panniculus carnosus layer to heal the wound by contraction. The wound healing process in humans is related to re-epithelialization and granulation tissue formation. Like collagen formation, normal tissue in rodents has a reticular collagen structure, whereas scar tissue has large parallel bundles of collagen arranged at approximately right angles to the basement membrane. In humans, instead of a random basketweave formation of the collagen fibers found in normal tissue, scars contain collagen that forms cross-links to align in a single direction parallel to the skin, opposite to the rat. In addition, scars have greater collagen density and larger fiber size compared to normal tissue (Whitby and Ferguson, 1991; Armour et al., 2007; Wolfram et al., 2009). In the future, we will consider moving to a porcine model to mimic more closely large wounds of the human body.

In conclusion, this study demonstrates a novel approach to treating large deep burn wounds by functionalizing endothelial cells with LDS on a collagen scaffold. For future studies, we intend to create large deep burn wounds on a porcine model to mimic human skin and further explore applications for LDS. At the same time, exploring the mechanism of LDS in different healing phases, in its interaction with other cell types, and how it regulates scar formation will be of clinical interest.

## 5 Conclusion

This study provides a promising novel treatment to accelerate large deep burn wound healing, thereby potentially reducing the morbidity of open burn wounds, such as insensible fluid losses and infection. Moreover, our scaffolds present the potential for treating large areas of deep burns by reducing and potentially obviating the need for autografting and its accompanying morbidity in patients with already limited areas of harvestable skin.

## Data Availability

The raw data supporting the conclusion of this article will be made available by the authors, without undue reservation.
